# Left Ventricular Wall Stress Is Sensitive Marker of Hypertrophic Cardiomyopathy With Preserved Ejection Fraction

**DOI:** 10.3389/fphys.2018.00250

**Published:** 2018-03-28

**Authors:** Xiaodan Zhao, Ru-San Tan, Hak-Chiaw Tang, Soo-Kng Teo, Yi Su, Min Wan, Shuang Leng, Jun-Mei Zhang, John Allen, Ghassan S. Kassab, Liang Zhong

**Affiliations:** ^1^National Heart Research Institute Singapore, National Heart Centre Singapore, Singapore, Singapore; ^2^Duke-NUS Medical School, Singapore, Singapore; ^3^Institute of High Performance Computing, Agency for Science, Technology and Research, Singapore, Singapore; ^4^School of Information Engineering, Nanchang University, Nanchang, Jiangxi, China; ^5^California Medical Innovations Institute, San Diego, CA, United States

**Keywords:** regional curvedness, regional wall stress index, regional area strain, hypertrophic cardiomyopathy, magnetic resonance imaging

## Abstract

Hypertrophic cardiomyopathy (HCM) patients present altered myocardial mechanics due to the hypertrophied ventricular wall and are typically diagnosed by the increase in myocardium wall thickness. This study aimed to quantify regional left ventricular (LV) shape, wall stress and deformation from cardiac magnetic resonance (MR) images in HCM patients and controls, in order to establish superior measures to differentiate HCM from controls. A total of 19 HCM patients and 19 controls underwent cardiac MR scans. The acquired MR images were used to reconstruct 3D LV geometrical models and compute the regional parameters (i.e., wall thickness, curvedness, wall stress, area strain and ejection fraction) based on the standard 16 segment model using our in-house software. HCM patients were further classified into four quartiles based on wall thickness at end diastole (ED) to assess the impact of wall thickness on these regional parameters. There was a significant difference between the HCM patients and controls for all regional parameters (*P* < 0.001). Wall thickness was greater in HCM patients at the end-diastolic and end-systolic phases, and thickness was most pronounced in segments at the septal regions. A multivariate stepwise selection algorithm identified wall stress index at ED (σ_*i,ED*_) as the single best independent predictor of HCM (AUC = 0.947). At the cutoff value σ_*i,ED*_ < 1.64, both sensitivity and specificity were 94.7%. This suggests that the end-diastolic wall stress index incorporating regional wall curvature—an index based on mechanical principle—is a sensitive biomarker for HCM diagnosis with potential utility in diagnostic and therapeutic assessment.

## Introduction

Hypertrophic cardiomyopathy (HCM) is a primary and familial disease of the cardiac sarcomere leading to cardiac hypertrophy (Kovacic and Muller, 2003; Hansen and Merchant, 2007). It is characterized by thickening of the myocardium with prevalence of 1 in 500 for the general population (Wigle, 2001; Kovacic and Muller, 2003; Elliott and McKenna, 2004; Hughes, 2004). Annual mortality is estimated at 1–2% (Wigle, 2001). Echocardiography can be used to measure ventricular thickness and diagnose hypertrophy (Klues et al., 1995; Maron, 2002). In addition, abnormal left ventricular (LV) systolic performance can also be detected and quantified by strain parameters (longitudinal strains and twist), and torsion and dyssynchrony (Carasso et al., 2008, 2010). There are still limitations to this method, however, as echocardiogram examination can be inconclusive when the hypertrophied myocardium is localized at LV regions that are difficult to visualize. Moreover, echocardiography may underestimate the maximum extent of LV wall thickening, particularly when hypertrophy involves the anterolateral wall (Maron et al., 2010). Compared to echocardiography, cardiac magnetic resonance (CMR) has the advantages of superior spatial resolution and ability to characterize tissue composition (Hoey et al., 2014) and ventricular shape (Zhong et al., 2009b, 2012b). Therefore, it provides opportunity for more accurate characterization of LV hypertrophy in HCM, both regionally and globally (Rickers et al., 2005; Noureldin et al., 2012; Lee et al., 2014).

The alteration of ventricular wall stress is associated with morphological and functional changes in the myocardium. LV wall stress is proportional to radius and inversely proportional to wall thickness according to the Law of Laplace (Badeer, 1963). Numerous formulas have been proposed to estimate wall stress (Falsetti et al., 1970; Grossman et al., 1975; Yin, 1981; Janz, 1982; Regen, 1990; Zhong et al., 2012a). Some early approaches assumed the heart as an ideal shape, such as spherical or ellipsoidal, which may not be applicable for complex LV geometry, such as in HCM. Moreover, they only allowed the global wall stress calculation, while 3D regional patterns and distributions of wall stress are crucial in fully characterizing, quantifying, and differentiating HCM patients from healthy subjects. We have proposed a 3D regional curvature-based wall stress approach and applied in ischemic dilated cardiomyopathy (Zhong et al., 2009b) and heart failure (Zhong et al., 2011). The pattern of HCM is variable and can be divided into morphological subtypes: reverse curvature, sigmoid and neutral. That may be associated with differential regional stress. Hence, appropriate characterization of regional morphology and wall stress may be particularly helpful in HCM.

In this study, we aimed to (1) assess the regional variation of wall curvedness, stress and function in HCM; (2) assess the utility of wall stress in differentiating HCM from controls, and (3) characterize the wall curvedness, stress and function in subtypes of HCM.

## Materials and methods

### Population

The study was approved by the SingHealth Centralized Institutional Review Board, and written consent forms were obtained from all participants. 19 HCM patients and 19 age-matched normal controls were prospectively enrolled at National Heart Centre Singapore. Subjects with LV ejection fraction <50%, hyperlipidemia, physician diagnosis of hypertension or diabetes mellitus were excluded from the Control group. Clinical data were collected at enrollment.

### CMR scan and LV wall thickness measurements

CMR scan was performed using steady state, free precession (SSFP) cine gradient echo sequences on a 1.5T Siemens MR imaging system (Avanto, Germany). Ventricular long axis (two-, three- and four-chamber) and stacks of short axis views with thickness 8 mm were each acquired in a single breath-hold. LV interventricular septum thickness in diastole (IVSd) and systole (IVSs), and LV posterior wall thicknesses in diastole (LVPWd) and systole (LVPWs) were measured from mid LV short-axis images, i.e., at the level of the papillary muscles.

### HCM subtypes

Following the HCM subtype characterization described by Binder et al. (2006), HCM cases were sub-categorized by our senior HCM consultant as sigmoid (*n* = 6), reverse curvature (*n* = 8) or neutral (*n* = 5).

### Two-dimensional regional curvature and strain

**Figure 1:**
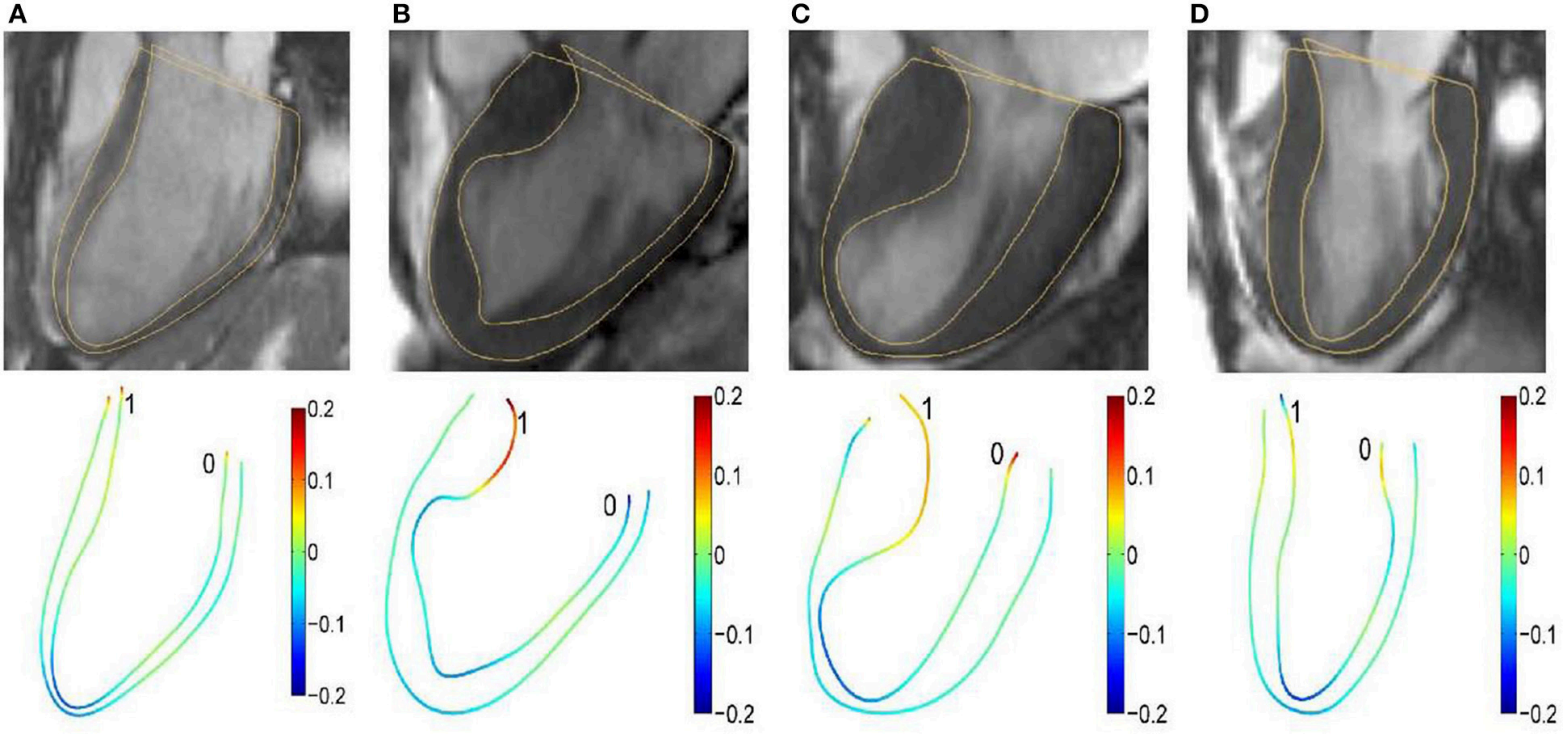
Segmented two-dimensional three-chamber long-axis magnetic resonance images (top row) and color representation of 2D curvature with range [−0.2, 0.2] (bottom row) in **(A)** a 37-year-old female control subject; **(B)** a 67-year-old female patient; **(C)** a 60-year-old female patient; and **(D)** a 55-year-old female patient. The curvature is negative if the unit tangent rotates clockwise.

A common approach to quantifying concavity and convexity of a contour employs curvature. The independent coordinate method (Lewiner et al., 2005; Zhao et al., 2018) has been used to compute endocardium and epicardium curvatures at both end-diastolic (ED) and end-systolic (ES) phases. Examples involving a control and three HCM subtypes at the ED phase are illustrated in Figure 1.

The extent of inhomogeneity was characterized by variation of curvature (*VC*) defined as the ratio of curvature standard deviation σ(κ) to mean μ(κ) at a given discrete point:

(1) $$\begin{array}{l} VC=\frac{\sigma \left(\kappa \right)}{\mu \left(\kappa \right)}. \end{array}$$

Ventricular endocardial and epicardial strain was defined as Zhao et al. (2018):

(2) $$\begin{array}{l} \begin{array}{c} S_{\text{endo}}=|\text{ln}\left(\frac{L_{\text{ES},\text{endo}}}{L_{\text{ED},\text{endo}}}\right)|\times 100\%;\\ S_{\text{epi}}=|\text{ln}\left(\frac{L_{\text{ES},\text{epi}}}{L_{\text{ED},\text{epi}}}\right)|\times 100\%, \end{array} \end{array}$$

where *L*_ED,endo_ and *L*_ED,epi_ are respective endocardial and epicardial contour lengths extending from one atrioventricular junction point to the other atrioventricular junction point in standard long axis view at ED, and similarly for *L*_ES,endo_ and *L*_ES,epi_.

### Three-dimensional regional shape and deformation parameters

LV endocardial and epicardial contours were segmented using CMRtools (Cardiovascular Solution, UK) by co-registering the short- and long-axis images. The segmented short-axis contours representing the endocardial and epicardial surfaces were then used as input for our 3D reconstruction algorithm. Our 3D reconstruction methodology can be broadly summarized into 3 steps:

(I) Correction of any possible motion artifacts due to respiration and patient movement. Here, we implement a shape-driven algorithm based on the premise that the LV epicardial surface must be smooth after the restoration process. This restoration is achieved by iterative in-plane translation of both the LV epicardial and endocardial contour vertices via minimization of an objective function based on the principal curvatures of the LV epicardial surface. Further details of the restoration algorithm can be found in our previous publications (Tan et al., 2013; Su et al., 2014).

(II) Up-sampling of short-axis contours and surface triangulation. The up-sampling of both the endocardial and epicardial contours are necessary to achieve a smooth reconstructed 3D surface, due to the relatively large spacing between the CMR image slices (typically 8–10 mm). We implement a B-spline fitting algorithm across multiple short-axis contours based on the surface normal vectors of the contour vertices to insert 3 intermediate points between any 2 adjacent contour vertices lying on the original CMR image slices. This results in the insertion of 3 intermediate short-axis contours between any 2 adjacent segmented contours. Next, we triangulate the up-sampled contours by connecting the 3 nearest points into a surface triangle. This step is repeated for each time-frame in the cardiac cycle and results in a set of 3D surface meshes with different number of vertices and triangles dependent on the height of the LV.

(III) Generation of endocardial surface meshes with 1-to-1 correspondence based on radial basis function morphing for the entire cardiac cycle to facilitate analysis of geometrical features. Here, we implement an automated approach to motion registered a series of surface meshes representing the instantaneous shape of the LV endocardial surface throughout the cardiac cycle from Step (II). The output is a sequence of meshes with 1-to-1 surface point correspondence; i.e., this sequence of meshes have identical number of vertices and the same connectivity information. Further details of the 1-to-1 correspondence algorithm can be found in our previous publication (Su et al., 2015). We note that this correspondence is implemented only for the LV endocardial surface meshes because our analysis focuses only on the curvature of the LV endocardial surface.

The format of the resultant endocardial surface is an explicit surface mesh in the form of a two-manifold structured triangle mesh where the vertices and connectivity information are stored.

The endocardial mesh was partitioned according to recommendation by the American Heart Association (Cerqueira et al., 2002). In this study, we used our modified approach (Zhong et al., 2009b; Su et al., 2012) to generate the 16-segment model, and omitted segment 17 in the standard nomenclature because the curvature of the true apex position would strain the reconstruction algorithm.

Wall thickness was evaluated for each segment at the ED and ES phases and denoted as *WT*_ED_ and *WT*_ES_, respectively (Zhong et al., 2009b). The maximal LV wall thickness among 16 segments at ED and ES were denoted as *WT*_ED,max_ and *WT*_ES,max_. The 3D regional shape was measured by the curvedness value *C* (Koenderink and Van Doorn, 1992) defined as:

(3) $$\begin{array}{l} C=\sqrt{\frac{\kappa_{1}^{2}+\kappa_{2}^{2}}{2},} \end{array}$$

where κ_1_ and κ_2_ are the maximum and minimum principal curvatures, respectively. These principal curvature are defined based on the endocardial surface. In the vicinity of any vertices on the endocardial surface mesh, the local surface can be approximated by an osculating paraboloid that may be represented by a quadratic polynomial. The detailed derivation for computing the principal curvatures can be found in Appendix A of our previous publications (Yeo et al., 2009; Zhong et al., 2009b).

**Figure 2:**
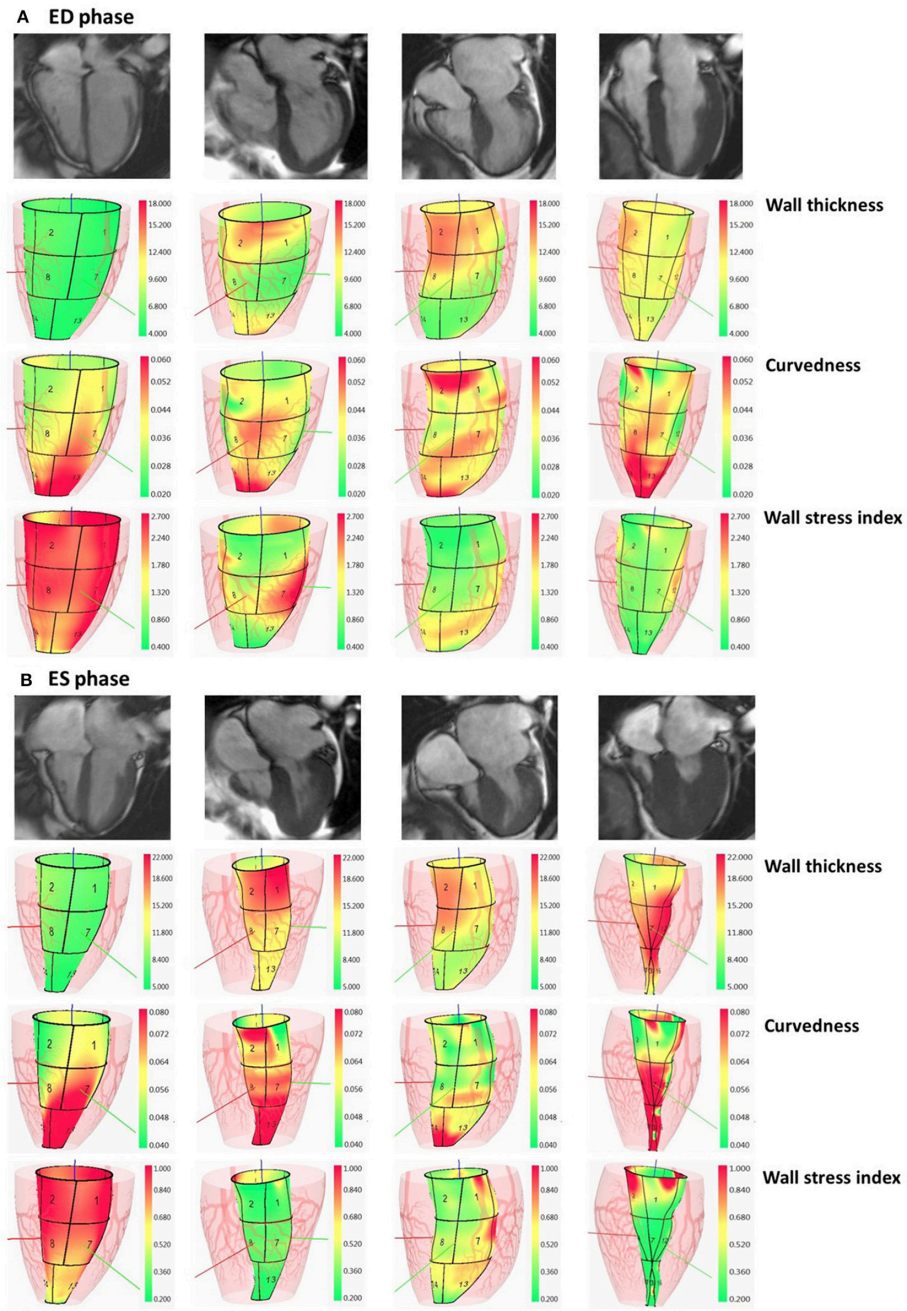
Columns: normal subject, HCM patient with sigmoid subtype, HCM patient with reverse curvature subtype and HCM patient with neutral subtype. In **(A)**, first row: segmented two-dimensional cine four-chamber magnetic resonance images at ED phase; second row: wall thickness (range: 4–18 mm) at ED phase; third row: regional curvedness (range: 0.02–0.06 mm^−1^) at ED phase; last row: wall stress index (range: 0.4–2.7) at ED phase. The order in **(B)** at ES phase is the same as the order in **(A)** with wall thickness range: 5–22 mm, regional curvedness range: 0.04–0.08 mm^−1^ and wall stress index range: 0.2–1.0. HCM, hypertrophic cardiomyopathy; ED, end diastole; ES, end systole.

Pressure-normalized wall stress, an index that provides crucial information on geometrical influence on wall stress, has been proposed using thick-walled ellipse and sphere models (Zhong et al., 2006; Alter et al., 2007). In the present study, wall stress index σ_*i*_, which incorporates local wall curvature, was determined as Zhong et al. (2009b)

(4) $$\begin{array}{l} \sigma_{i}=\frac{R}{2WT\left(1+\frac{WT}{2R}\right)}, \end{array}$$

where *WT* is ventricular wall thickness and *R* the inner radius of curvedness. In Figure 2, we illustrate wall thickness, regional curvedness and wall stress index for a control patient, sigmoid, reverse curvature and neutral subtypes for HCM patients.

Area strain is a dimensionless quantity that measures regional LV endocardial surface deformation which integrates longitudinal, circumferential and radial deformation. The regional area strain (*AS*) was defined as Zhong et al. (2012b):

(5) $$\begin{array}{l} AS=\text{ln}\left(\frac{SA_{ES}}{SA_{ED}}\right), \end{array}$$

where *SA*_ED_ and *SA*_ES_ are the respective endocardial surface areas at the ED and ES phases.

The regional ejection fraction (*EF*) is a measure of the pumping efficiency of a particular LV segment and was previously derived (Wisneski et al., 1981; Teo et al., 2015). The *EF*_*i*_ for the i-th segment is calculated as

(6) $$\begin{array}{l} EF_{i}=\frac{V_{i,ED}-V_{i,ES}}{V_{i,ED}}\times 100\%, \end{array}$$

where *V*_*i*, ED_ and *V*_*i*, ES_ are the LV cavity volumes corresponding to the i-th segment at the ED and ES phase, respectively.

### Statistical analysis

All continuous variables are presented as mean ± standard deviation (SD), whereas categorical data are presented as relative frequencies in terms of percentage. Associations between continuous variables were investigated using least square regression and Pearson correlation. The two-sample *t-*test was used to assess significant differences between means of two independent groups. One-way analysis of variance (ANOVA) was used to compare means among control and hypertrophy subtypes for 3D regional parameters. As individual diagnostic cutpoints, the continuous predictors IVSd, *WT*_ED,max_ and σ_*i,ED*_ were dichotomized (non-HCM vs. HCM) as follows: IVSd ≤13 mm vs. >13 mm, *WT*_ED,max_ ≤ 13 mm vs. >13 mm, and σ_*i,ED*_ ≥1.64 vs. <1.64. Potential predictors were assessed individually using univariate logistic regression and those significant at *P* < 0.20 were included in a multivariate analysis incorporating a stepwise selection algorithm (SLE = 0.20, SLS = 0.25) to identify a minimal “best” subset predictive of HCM. Intra- and inter-observer reproducibility was assessed via the intra-class correlation coefficient (ICC). The mean of the absolute values of the differences between two measurements divided by the mean of all measurements taken was used to quantify measurement variability as a proportion of the mean measurement value (Zhong et al., 2009a, 2012c). *P* < 0.05 was considered statistically significant. Data assembly and statistical analysis were performed with SPSS version 22.0.

## Results

The baseline demographics of controls and HCM patient are summarized in Table 1. Compared to control subjects, HCM patients had higher LV end-diastolic and lower end-systolic volume indices, although not statistically significant (*P* = 0.451 and *P* = 0.308); however, difference in higher LV ejection fraction was statistically significant (*P* = 0.034). For 2D clinical CMR measurements, wall thickness was demonstrably greater in HCM patients vs. controls for ED (HCM, 17.0 ± 6.1 vs. Control, 8.4 ± 1.4 mm; *P* < 0.001), ES (HCM, 21.4 ± 5.4 vs. Control, 12.2 ± 2.3 mm; *P* < 0.001) and fractional shortening (HCM, 44.2 ± 7.9 vs. Control, 35.0 ± 6.1%; *P* < 0.001; Table 2). Similar results in ED and ES maximal wall thickness were observed between HCM patients and controls for CMR measurements from our 3D model.

**Table 1:** Baseline and demographics of control subjects and HCM patients.

**Variable**	**Control (*n* = 19)**	**HCM (n = 19)**	***P-*value**
Age, years old	51 ± 11	51 ± 13	0.834
Gender, Male/Female	12/7	7/12	0.105
Weight, kg	68 ± 15	69 ± 20	0.816
Height, cm	163 ± 11	162 ± 12	0.810
Body surface area, m^2^	1.75 ± 0.24	1.76 ± 0.30	0.953
Diastolic blood pressure, mmHg	74 ± 8	72 ± 13	0.732
Systolic blood pressure, mmHg	128 ± 17	132 ± 22	0.548
Tobacco, %	0 (0%)	2 (10.5%)	0.181
Diabetes, %	0 (0%)	1 (5.3%)	0.432
Hyperlipidaemia, %	0 (0%)	9 (47.4%)	**<0.001**
Hypertension, %	0 (0%)	7 (36.8%)	**0.001**
Peripheral vascular disease, %	0 (0%)	1 (5.3%)	0.432
Family history of HCM (up to second degree)	0 (0%)	9 (47.4%)	**<0.001**
Family history of sudden cardiac death due to HCM	0 (0%)	4 (21.1%)	**0.029**
LVEDV index, ml/m^2^	74 ± 12	77 ± 15	0.451
LVESV index, ml/m^2^	25 ± 8	22 ± 10	0.308
LV ejection fraction, %	66 ± 6	72 ± 9	**0.034**
LV mass index, g/m^2^	54 ± 10	101 ± 43	**<0.001**

*Data are expressed as mean ± SD or as number (percentage). HCM, hypertrophic cardiomyopathy; LVEDV, left ventricle end diastolic volume; LVESV, left ventricle end systolic volume; LV, left ventricle. Bold values mean statistically significant*.

**Table 2:** Comparison of wall thickness between 2D clinical and 3D model measurements.

**Variable**	**Control (*n* = 19)**	**HCM (*n* = 19)**	***P-*value**
**2D CLINICAL MEASUREMENTS**			
IVSd, mm	8.4 ± 1.4	17.0 ± 6.1	<**0.001**
IVSs, mm	12.2 ± 2.3	21.4 ± 5.4	<**0.001**
LVPWd, mm	6.1 ± 1.4	8.8 ± 3.4	**0.009**
LVPWs, mm	12.7 ± 2.3	18.5 ± 5.4	<**0.001**
FS, %	35.0 ± 6.1	44.2 ± 7.9	<**0.001**
**3D MODEL MEASUREMENTS**			
*WT*_ED,max_, mm	8.1 ± 1.4	16.5 ± 5.2	**0.001**
*WT*_ES,max_, mm	12.4 ± 1.5	22.1 ± 5.0	<**0.001**

*Data are expressed as mean ± SD. HCM, hypertrophic cardiomyopathy; IVSd (IVSs), interventricular septum in diastole (systole); LVPWd (LVPWs), left ventricular posterior wall in diastole (systole); FS, fractional shortening. WT_ED,max_ (WT_ES,max_), maximal wall thickness among 16 regional segments in diastole (systole) from 3D model. Bold values mean statistically significant*.

### Difference of geometrical descriptors between HCM and controls

**Figure 3:**
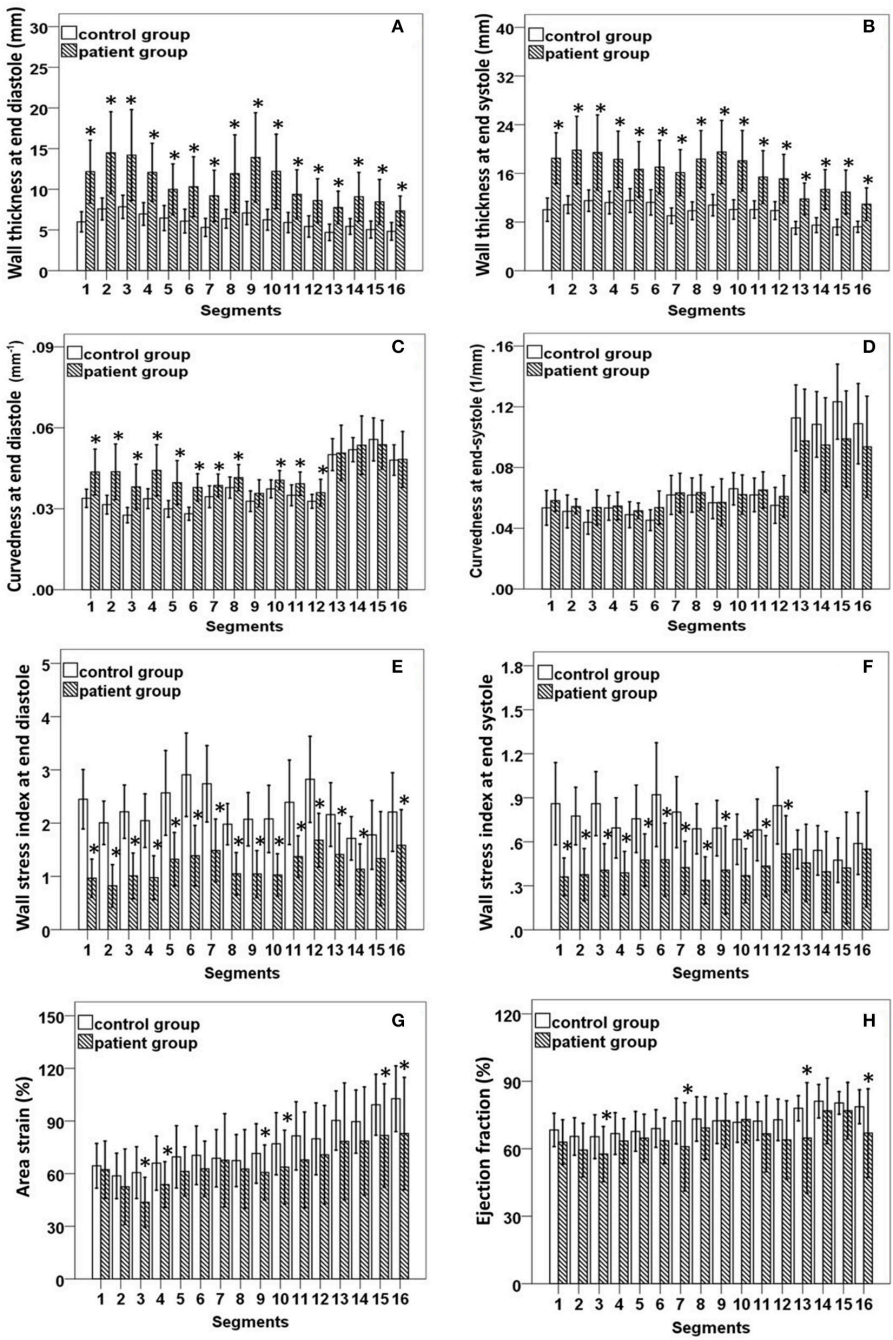
**(A,B)** Comparison of wall thickness at end diastole (left) and end systole (right); **(C,D)** Comparison of curvedness at end diastole (left) and end systole (right); **(E,F)** Comparison of wall stress index at end diastole (left) and end systole (right); **(G,H)** Comparison of area strain (left) and ejection fraction (right) between control group and patient group with hypertrophic cardiomyopathy. *Significant difference between two groups (*P* < 0.05).

The 2D curvature and strain results for both groups are summarized in Table 3. The *VC* for curvature was 1.94 ± 0.47 at ED and 3.54 ± 1.32 at ES in the controls, with increases in HCM patients to 2.65 ± 0.84 at ED and 5.02 ± 2.42 at ES. Second, HCM patients had significantly lower *S*_endo_ and *S*_epi_ compared with controls (18.4 ± 3.8% vs. 24.8 ± 3.0% and 12.6 ± 3.9% vs. 21.3 ± 3.0%, both *P* < 0.001). Patients with HCM had significant increases in wall thickness at both ED and ES phases (Figures 3A,B). For each region in HCM patients, greater wall thickness was observed in the basal anterior septal, basal inferior septal, mid inferior septal and apical septal regions owing to septum hypertrophy.

**Table 3:** Variation of 2D curvature, length and strains for controls and HCM patients.

**Variable**	**Control (*n* = 19)**	**HCM (*n* = 19)**	***P*-value**
Variation of curvature at ED phase	1.94 ± 0.47	2.65 ± 0.84	**0.003**
Variation of curvature at ES phase	3.54 ± 1.32	5.02 ± 2.42	**0.026**
ED endocardial length, mm	125.8 ± 16.4	124.1 ± 14.5	0.740
ED epicardial length, mm	132.2 ± 17.5	131.9 ± 18.0	0.960
ES endocardial length, mm	98.2 ± 13.5	103.6 ± 14.5	0.246
ES epicardial length, mm	107.0 ± 14.7	116.7 ± 19.1	0.087
*S*_endo_, %	24.8 ± 3.0	18.4 ± 3.8	**<0.001**
*S*_epi_, %	21.3 ± 3.0	12.6 ± 3.9	**<0.001**

*Data are expressed as mean ± SD. HCM, hypertrophic cardiomyopathy; ED, end diastole; ES, end systole; S_endo_ (S_epi_), average endocardial (epicardial) strain of 2-, 3-, and 4-chamber endocardial (epicardial) strain. Bold values mean statistically significant*.

Regional 3D curvedness, regional wall stress index, area strain and ejection fraction are given in Tables 4–6, respectively. Figures 3C,D showed a significant increase in regional ED curvedness in HCM patients compared to controls, except for segment 9 (mid inferior septal) and segments 13-16 (apical region). Figures 3E,F showed a significant decrease in wall stress index at ED and ES in the HCM patients across all segments, except for segment 15 (apical inferior) at ED and segments 13–16 (apical region) at ES. Figure 3G demonstrated a decrease in *AS* in HCM patients. Mean *AS* values (aggregating over all 16 segments) were 78.5 and 65.7% for controls and HCM patients respectively (*P* < 0.05). Comparing across individual segments, only the inferior regions (segments 3–5, 9–11, and 15) and the apical lateral segment (segment 16) exhibited significant differences. Mean *EF* values (aggregating over all 16 segments) were 72.2 and 66.5% for controls and HCM patients, respectively. Comparing across individual segments, significant differences were observed for segments 3 (basal inferior septal), 7 (mid anterior), 13 (apical anterior) and 16 (apical lateral) (Figure 3H).

**Table 4:** Curvedness computed from the 3-D reconstructed model of the LV at end diastole and end systole for Controls and HCM patients.

**Segment**	**Curvedness at end diastole (mm**^**−1**^**)**		**Curvedness at end systole (mm**^**−1**^**)**	
	**Control (*n* = 19)**	**HCM (*n* = 19)**	**Control (*n* = 19)**	**HCM (*n* = 19)**
(1) basal anterior	0.0339 ± 0.0034	0.0436 ± 0.0085^*^	0.0535 ± 0.0114	0.0582 ± 0.0072
(2) basal anterior septal	0.0315 ± 0.0035	0.0437 ± 0.0103^*^	0.0511 ± 0.0108	0.0543 ± 0.0049
(3) basal inferior septal	0.0276 ± 0.0028	0.0381 ± 0.0084^*^	0.0439 ± 0.0078	0.0537 ± 0.0116^*^
(4) basal inferior	0.0337 ± 0.0037	0.0442 ± 0.0095^*^	0.0534 ± 0.0081	0.0546 ± 0.0091
(5) basal inferior lateral	0.0300 ± 0.0031	0.0397 ± 0.0082^*^	0.0489 ± 0.0085	0.0515 ± 0.0051
(6) basal anterior lateral	0.0282 ± 0.0024	0.0379 ± 0.0051^*^	0.0453 ± 0.0069	0.0536 ± 0.0110^*^
(7) mid anterior	0.0345 ± 0.0040	0.0386 ± 0.0042^*^	0.0619 ± 0.0128	0.0633 ± 0.0130
(8) mid anterior septal	0.0378 ± 0.0039	0.0415 ± 0.0048^*^	0.0619 ± 0.0113	0.0635 ± 0.0116
(9) mid inferior septal	0.0328 ± 0.0038	0.0357 ± 0.0050	0.0568 ± 0.0105	0.0570 ± 0.0155
(10) mid inferior	0.0374 ± 0.0033	0.0406 ± 0.0035^*^	0.0660 ± 0.0106	0.0622 ± 0.0129
(11) mid inferior lateral	0.0350 ± 0.0039	0.0393 ± 0.0043^*^	0.0620 ± 0.0110	0.0652 ± 0.0120
(12) mid anterior lateral	0.0327 ± 0.0026	0.0359 ± 0.0050^*^	0.0551 ± 0.0118	0.0610 ± 0.0137
(13) apical anterior	0.0500 ± 0.0059	0.0507 ± 0.0103	0.1127 ± 0.0217	0.0975 ± 0.0341
(14) apical septal	0.0519 ± 0.0045	0.0536 ± 0.0108	0.1084 ± 0.0216	0.0949 ± 0.0311
(15) apical inferior	0.0557 ± 0.0079	0.0537 ± 0.0090	0.1233 ± 0.0247	0.0989 ± 0.0316^*^
(16) apical lateral	0.0480 ± 0.0057	0.0483 ± 0.0103	0.1089 ± 0.0264	0.0937 ± 0.0333
Mean	0.0375 ± 0.0095	0.0428 ± 0.0095^*^	0.0696 ± 0.0299	0.0677 ± 0.0251

*Data are expressed as mean ± SD. HCM, hypertrophic cardiomyopathy; LV, left ventricle*.

* *Significant difference between control subjects and HCM patients (P < 0.05)*.

**Table 5:** Wall stress index computed from the 3-D reconstructed model of the LV at end diastole and end systole for Controls and HCM patients.

**Segment**	**Wall stress index at end diastole**		**Wall stress index at end systole**	
	**Control (*n* = 19)**	**HCM (*n* = 19)**	**Control (*n* = 19)**	**HCM (*n* = 19)**
(1) basal anterior	2.45 ± 0.56	0.97 ± 0.36^*^	0.86 ± 0.28	0.36 ± 0.13^*^
(2) basal anterior septal	2.01 ± 0.41	0.82 ± 0.40^*^	0.78 ± 0.20	0.38 ± 0.18^*^
(3) basal inferior septal	2.21 ± 0.50	1.01 ± 0.43^*^	0.86 ± 0.22	0.41 ± 0.18^*^
(4) basal inferior	2.05 ± 0.50	0.98 ± 0.41^*^	0.69 ± 0.21	0.39 ± 0.15^*^
(5) basal inferior lateral	2.57 ± 0.79	1.32 ± 0.50^*^	0.76 ± 0.23	0.48 ± 0.18^*^
(6) basal anterior lateral	2.91 ± 0.78	1.39 ± 0.57^*^	0.92 ± 0.35	0.48 ± 0.25^*^
(7) mid anterior	2.74 ± 0.72	1.49 ± 0.59^*^	0.80 ± 0.24	0.42 ± 0.18^*^
(8) mid anterior septal	1.98 ± 0.39	1.05 ± 0.40^*^	0.69 ± 0.17	0.34 ± 0.16^*^
(9) mid inferior septal	2.08 ± 0.50	1.05 ± 0.44^*^	0.69 ± 0.19	0.41 ± 0.30^*^
(10) mid inferior	2.08 ± 0.63	1.03 ± 0.40^*^	0.62 ± 0.17	0.37 ± 0.18^*^
(11) mid inferior lateral	2.39 ± 0.79	1.37 ± 0.39^*^	0.68 ± 0.21	0.43 ± 0.21^*^
(12) mid anterior lateral	2.82 ± 0.81	1.68 ± 0.50^*^	0.85 ± 0.26	0.52 ± 0.26^*^
(13) apical anterior	2.16 ± 0.60	1.41 ± 0.58^*^	0.55 ± 0.13	0.46 ± 0.26
(14) apical septal	1.71 ± 0.41	1.13 ± 0.47^*^	0.54 ± 0.17	0.40 ± 0.28
(15) apical inferior	1.78 ± 0.65	1.33 ± 0.88	0.47 ± 0.15	0.42 ± 0.38
(16) apical lateral	2.21 ± 0.74	1.58 ± 0.67^*^	0.59 ± 0.21	0.55 ± 0.39
Mean	2.45 ± 0.56	0.97 ± 0.36^*^	0.86 ± 0.28	0.36 ± 0.13^*^

*Data are expressed as mean ± SD. HCM, hypertrophic cardiomyopathy; LV, left ventricle*.

* *Significant difference between control subjects and HCM patients (P < 0.05)*.

**Table 6:** Area strain (%) and ejection fraction (%) computed from the 3-D reconstructed model of the LV for control and HCM patients.

**Segment**	**Area strain (%)**		**Ejection fraction (%)**	
	**Control (*n* = 19)**	**HCM (*n* = 19)**	**Control (*n* = 19)**	**HCM (*n* = 19)**
(1) basal anterior	66.5 ± 12.9	62.3 ± 16.3	68.4 ± 7.4	63.0 ± 9.9
(2) basal anterior septal	60.7 ± 13.5	52.5 ± 21.5	65.5 ± 8.3	59.5 ± 11.9
(3) basal inferior septal	62.5 ± 14.1	43.7 ± 14.2^*^	65.4 ± 9.7	57.6 ± 12.3^*^
(4) basal inferior	68.0 ± 15.0	53.8 ± 13.0^*^	66.7 ± 9.3	63.4 ± 10.0
(5) basal inferior lateral	72.4 ± 17.6	61.2 ± 14.0^*^	67.7 ± 8.9	64.7 ± 10.6
(6) basal anterior lateral	73.4 ± 16.5	62.8 ± 15.8	69.0 ± 8.3	63.6 ± 10.1
(7) mid anterior	71.6 ± 18.0	67.7 ± 26.5	72.3 ± 10.1	60.9 ± 19.6^*^
(8) mid anterior septal	70.1 ± 16.8	62.7 ± 22.4	73.2 ± 9.9	69.2 ± 13.9
(9) mid inferior septal	73.9 ± 17.5	60.7 ± 15.7^*^	72.4 ± 10.1	72.5 ± 11.9
(10) mid inferior	79.9 ± 18.4	63.7 ± 21.1^*^	71.8 ± 8.9	73.0 ± 10.3
(11) mid inferior lateral	84.6 ± 20.2	67.8 ± 27.3^*^	72.3 ± 8.5	66.7 ± 16.9
(12) mid anterior lateral	82.5 ± 21.2	70.8 ± 28.1	72.9 ± 9.2	63.9 ± 17.4
(13) apical anterior	92.2 ± 18.6	78.4 ± 33.3	78.0 ± 5.6	64.8 ± 24.6^*^
(14) apical septal	92.2 ± 20.6	78.6 ± 30.9	81.1 ± 7.5	76.9 ± 14.5
(15) apical inferior	101.4 ± 18.5	81.8 ± 29.4^*^	80.4 ± 5.0	76.9 ± 12.6
(16) apical lateral	104.7 ± 18.9	82.9 ± 31.9^*^	78.7 ± 7.6	67.0 ± 19.7^*^
Mean	78.5 ± 21.5	65.7 ± 25.2^*^	72.2 ± 9.7	66.5 ± 15.5^*^
**AGGREGATING OVER THE BASAL, MID-CAVITY AND APICAL REGIONS**				
(i) basal	67.2 ± 14.3	56.0 ± 14.4^*^	67.1 ± 8.3	62.0 ± 9.5
(ii) mid-cavity	77.1 ± 18.1	65.6 ± 22.8	72.5 ± 9.2	68.4 ± 11.8
(iii) apical	97.6 ± 18.1	80.4 ± 30.8^*^	79.5 ± 5.6	72.4 ± 15.3

*Data are expressed as mean ± SD. HCM, hypertrophic cardiomyopathy; LV, left ventricle*.

* *Significant difference between control subjects and HCM patients (P < 0.05)*.

### Difference of geometrical descriptors in HCM subtypes

According to the characterization of HCM morphological subtypes described in section HCM subtypes, our HCM group included sigmoid subtypes (*n* = 6, total of 6 × 16 = 96 segments), reverse curvature subtypes (*n* = 8, total of 8 × 16 = 128 segments) and neutral subtypes (*n* = 5, total of 5 × 16 = 80 segments). Results for all 3D regional parameters were given in Table 7. Compared with controls, all three subtypes had significantly thicker ventricular walls (*P* < 0.001), with wall thickness increasing from sigmoid to reverse curvature to neutral subtypes. Controls had significantly less curvature at ED and higher wall stress index compared to the three HCM subtype groups. The neutral subtype had the thickest ventricular wall and lowest wall stress index at ES compared to the other two HCM subtypes (all *P* < 0.001). The reverse curvature subtype had significantly lower *AS* and *EF* compared to the other two HCM subtypes.

**Table 7:** ANOVA analysis between control and hypertrophy subtypes for 3D regional parameters.

**Variable**	**Control (*n* = 304)**	**Sigmoid (*n* = 96)**	**Reverse Curvature (*n* = 128)**	**Neutral (*n* = 80)**	***P*-value**
*WT*_ED_, mm	6.08 ± 1.54	9.53 ± 3.76^*^^†^^‡^	11.15 ± 4.52^*^^†^	11.37 ± 4.47^*^^‡^	<**0.001**
*WT*_ES_, mm	9.69 ± 2.19	15.25 ± 4.54^*^^‡^	16.22 ± 5.60^*^^§^	17.81 ± 4.50^*^^‡^^§^	<**0.001**
*C*_ED_, mm^−1^	0.0375 ± 0.0095	0.0418 ± 0.0094^*^	0.0431 ± 0.0092^*^	0.0436 ± 0.0100^*^	<**0.001**
*C*_ES_, mm^−1^	0.0696 ± 0.0299	0.0707 ± 0.0251	0.0615 ± 0.0194^*^^§^	0.0741 ± 0.0305^§^	**0.005**
σ_*i,ED*_	2.26 ± 0.70	1.40 ± 0.54^*^^‡^	1.19 ± 0.62^*^	1.07 ± 0.41^*^^‡^	<**0.001**
σ_*i,ES*_	0.71 ± 0.25	0.43 ± 0.23^*^^‡^	0.49 ± 0.29^*^^§^	0.31 ± 0.10^*^^‡^^§^	<**0.001**
*AS*, %	78.51 ± 21.48	71.59 ± 22.11^†^	55.24 ± 20.82^*^^†^^§^	75.36 ± 29.45^§^	<**0.001**
*EF*, %	72.23 ± 9.66	70.49 ± 1.071^†^	61.03 ± 16.05^*^^†^^§^	70.38 ± 16.45^§^	<**0.001**

*Data are expressed as mean ± SD. WT_ED_, wall thickness at end diastole; WT_ES_, wall thickness at end systole; C_ED_, curvedness at end diastole; C_ES_, curvedness at end systole; σ_i,ED_, wall stress index at end diastole; σ_i,ES_, wall stress index at end systole; AS, area strain; EF, ejection fraction*.

* Significant differences between control group and three HCM subtypes;

† significant difference between sigmoid and reverse curvature subtypes;

‡ significant difference between sigmoid and neutral subtypes;

§ *significant difference between reverse curvature and neutral subtypes. Bold values mean statistically significant*.

### Univariate and multivariate analysis

**Figure 4:**
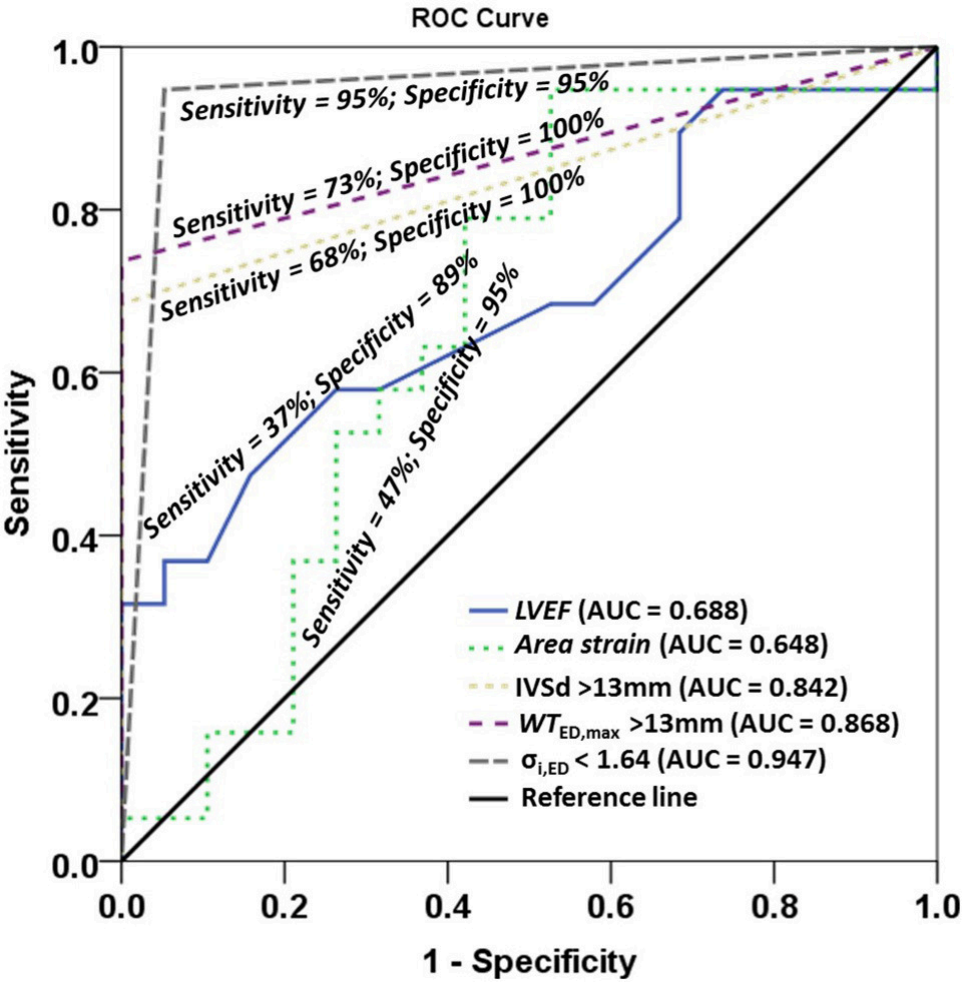
Receiver operating characteristic (ROC) curves for left ventricle ejection fraction (LVEF), area strain, and three dichotomized parameters IVSd >13mm, *WT*_ED,max_ >13 mm, σ_*i,ED*_ < 1.64. IVSd, interventricular septum in diastole from 2D clinical CMR measurement; *WT*_ED,max_, maximal wall thickness among 16 regional segments in diastole from 3D model; σ_*i,ED*_, wall stress index at end diastole.

Non-decompensated HCM patients tend to have normal LVEF. In seeking a better indicator for differentiating HCM patients, we performed univariate logistic regression analysis for LVEF, area strain, and three dichotomized parameters, viz., IVSd >13 mm, *WT*_ED,max_ >13 mm, σ_*i,ED*_ < 1.64. A multivariate stepwise selection algorithm (SLE = 0.20, SLS = 0.25) on the five variables significant at P < 0.20 in univariate analysis identified σ_*i,ED*_ < 1.64 as the single best independent predictor of HCM group (P < 0.001). Analysis results are given in Table 8, and ROC curves for the five parameters are plotted in Figure 4 with corresponding AUC, sensitivity and specificity. σ_*i,ED*_ < 1.64 exhibited the highest sensitivity (94.7%) and specificity (94.7%) for differentiating HCM patients from controls with AUC = 0.947.

**Table 8:** Univariate logistic regression and multivariate stepwise selection analysis.

**Variable**	***P*****-value**	
	**Univariate logistic regression analysis**	**Multivariate stepwise selection analysis**
LVEF, %	0.065	–
Area strain, %	0.0970	–
IVSd >13 mm	**0.005**	–
*WT*_ED,max_ >13 mm	**0.0031**	–
σ_*i,ED*_ < 1.64	<**0.001**	<**0.001**

*LVEF, left ventricular ejection fraction; IVSd, interventricular septum thickness in diastole from 2D clinical measurement; WT_ED,max_, maximal wall thickness among 16 segments in diastole from 3D model; σ_i,ED_, wall stress index at end diastole. Bold values mean statistically significant*.

### Impact of wall thickness on 3D geometrical descriptors

**Figure 5:**
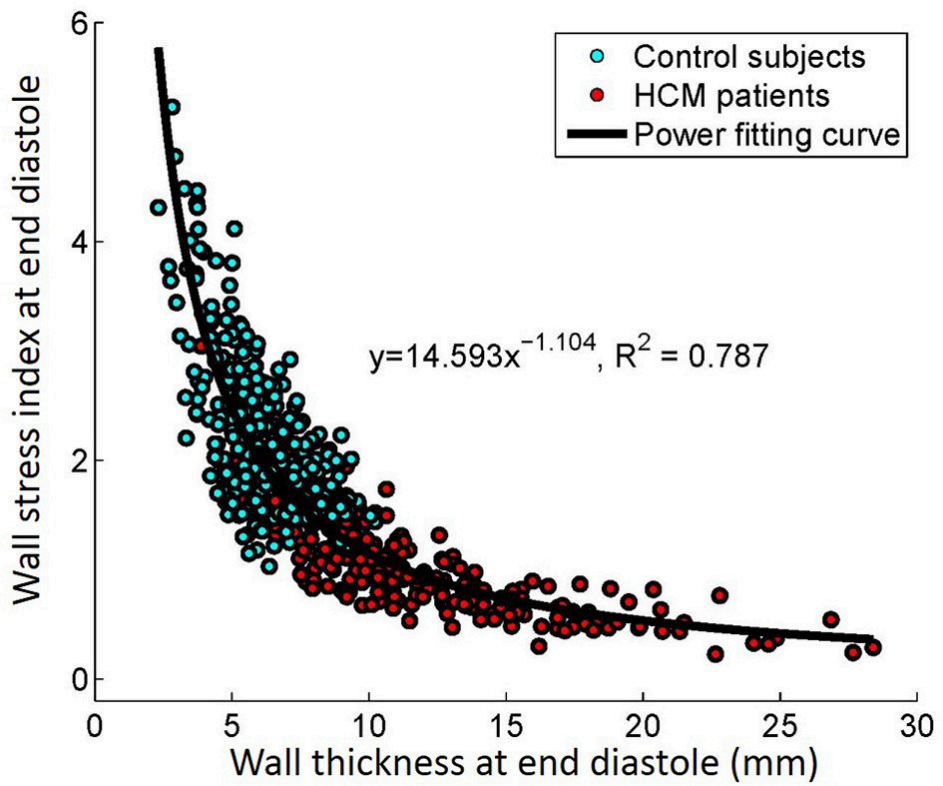
Correlation between wall thickness and wall stress index at end diastole.

**Figure 6:**
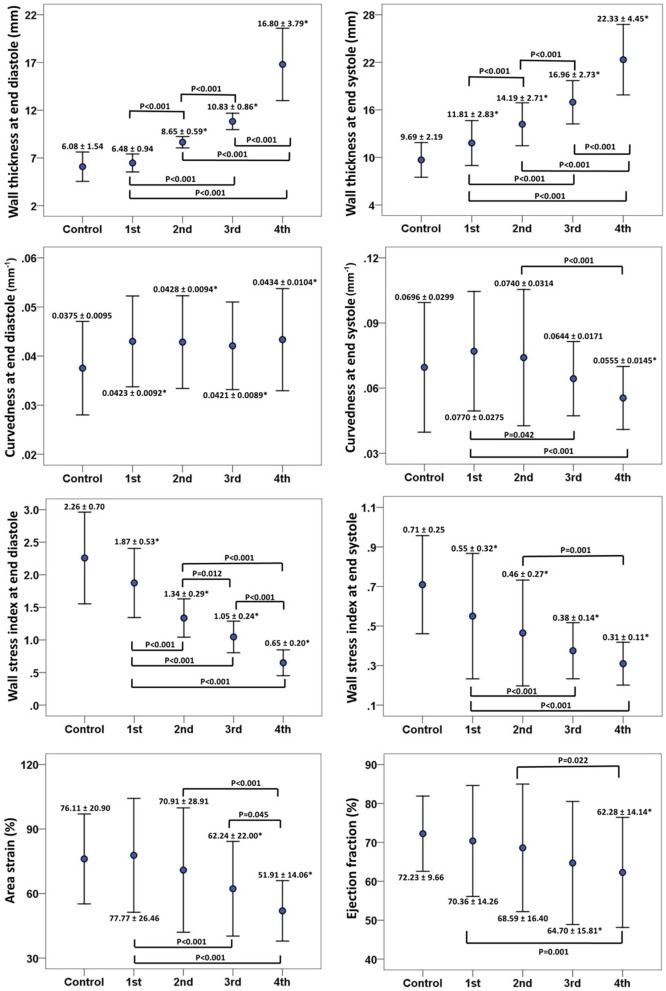
Error bar plots (mean ± SD) between control and quartiles divided by left ventricular end-diastolic wall thickness in patients with hypertrophy cardiomyopathy. First row: wall thickness at ED **(left)** and ES **(right)**, ED = end diastole; ES = end systole; second row: 3D regional curvedness at ED **(left)** and ES **(right)**; third row: wall stress index at ED **(left)** and ES **(right)**; last row: area strain **(left)** and ejection fraction **(right)**. *Significant difference between control group and four quartiles (*P* < 0.05).

There was inverse relationship between wall thickness and wall stress index ($\sigma_{i,ED}=14.593\times W{T_{ED}}^{-1.104},\;R^{2}=0.787,P<0.001$), as shown in Figure 5. To further investigate the impact of wall thickness on regional ventricular shape and function, we divided the HCM patients (consisting of 16 × 19 = 304 segments) into four quartiles based on wall thickness (mm) at ED: <7.72 mm, 7.72–9.63 mm, 9.63–12.68 mm and >12.68 mm in Figure 6. With increasing wall thickness, ventricular curvedness showed no significant change at ED, but decreased at ES. There was slightly augmented area strain in 1st quartile, but decreased from quartile 2–4. There was a reduction of ejection fraction, but not significant.

### Reproducibility

Intra-class correlation (ICC) with 95% CI, mean difference ± SD, and percentage variability (%) were computed for 5 control subjects and 5 HCM patients (10 × 16 = 160 segments) from intra- and inter-observer studies, and the reproducibility results were given in Table 9. All parameters were highly reproducible with ICC >0.87, and percentage variability was ≤6.5% for intra-observer and ≤5.0% for inter-observer.

**Table 9:** Intra- and inter-observer reproducibility in 5 control subjects and 5 HCM patients.

**Variable**	**Intra-class correlation coefficient (95% CI)**	**Mean difference ± SD**	**Percentage variability (%)**
**INTRA OBSERVER REPRODUCIBILITY**			
*WT*_ED_, mm	0.995 (0.993–0.996)	−0.050 ± 0.488	3.11
*WT*_ES_, mm	0.992 (0.989–0.994)	0.146 ± 0.733	2.41
*C*_ED_, mm^−1^	0.893 (0.856–0.920)	0.0020 ± 0.0044	4.66
*C*_ES_, mm^−1^	0.979 (0.972–0.985)	0.0001 ± 0.0057	3.50
σ_*i,ED*_	0.985 (0.979–0.989)	−0.049 ± 0.187	6.03
σ_*i,ES*_	0.926 (0.900–0.945)	0.008 ± 0.096	6.50
*AS*, %	0.930 (0.906–0.949)	1.889 ± 8.127	4.40
*EF*, %	0.885 (0.845–0.914)	0.445 ± 4.609	3.04
**INTER OBSERVER REPRODUCIBILITY**			
*WT*_ED_, mm	0.997 (0.996-0.998)	−0.019 ± 0.375	2.36
*WT*_ES_, mm	0.995 (0.993-0.996)	0.193 ± 0.574	1.69
*C*_ED_, mm^−1^	0.917 (0.888-0.938)	0.0019 ± 0.0039	4.21
*C*_ES_, mm^−1^	0.983 (0.977-0.988)	0.0006 ± 0.0051	2.77
σ_*i,ED*_	0.987 (0.982-0.990)	0.018 ± 0.160	4.99
σ_*i,ES*_	0.941 (0.921-0.957)	−0.002 ± 0.088	5.01
*AS*, %	0.928 (0.903-0.947)	2.853 ± 8.190	4.24
*EF*, %	0.879 (0.838-0.910)	0.949 ± 4.752	3.16

*HCM, hypertrophic cardiomyopathy; CI, confidence interval; SD, standard deviation; Percentage variability, the mean of the absolute values of the differences between two measurements divided by their mean; WT_ED_, wall thickness at ED; WT_ES_, wall thickness at ES; ED, end diastole; ES, end systole; C_ED_, curvedness at ED; C_ES_, curvedness at ES; σ_i,ED_, wall stress index at ED; σ_i,ES_, wall stress index at ES; AS, area strain; EF, ejection fraction*.

## Discussion

The main finding of this study was that curvedness-based ventricular wall stress index at end diastole (ED) was a more sensitive and specific parameter than traditional ventricular wall thickness and other measures for differentiating HCM with preserved ejection fraction. Furthermore, among the three HCM subtypes, the neutral group presented lowest wall stress, but reverse curvature group presented lowest regional contractile function compared to the control group and other two subtypes (*P* < 0.05).

### Ventricular wall stress measurement

In the present study, wall stress index is a pure geometric parameter that quantifies the physical response of left ventricle to loading and allows a comparison between ventricles under differing pressures. The wall stress index is expressed as the ratio of wall thickness to wall radius (*h*/*R*) which takes into account regional ventricular curvedness. We have demonstrated excellent intra- and inter-observer reproducibility in wall stress measurement for both normal and HCM patients with percentage variability less than 6%. This is in significant contrast to previous echocardiographic studies (i.e., 7–11%; Greim et al., 1995). This is likely to reflect the better accuracy of CMR to regional wall curvedness and thickness than echocardiography, which has been demonstrated in several previous studies examining different cardiac conditions (Zhong et al., 2009b, 2011, 2012b).

The joint use of imaging and modeling of the heart has opened up possibilities for a better thorough understanding and evaluation of the LV wall stress. Traditionally, most work on wall stress has been based on two-dimensional and three-dimensional models that are represented by simplified idealized geometry analyses with different formula (i.e., sphere, spheroid, ellipsoid; Yin, 1981; Zhong et al., 2006, 2007, 2012a). Finite element analysis (FEA), an engineering technique utilized to study complex structure, can overcome some of these limitations. Previous studies has elucidated the characteristics of wall stress and clarified how they should be properly analyzed so that these concepts can be applied in translational research (Guccione et al., 1995; Dorri et al., 2006; Lee et al., 2014; Choy et al., 2018). However, from the clinical application consideration, the application of FEA to employ human *in vivo* data still remain a challenge. Our approach allows precise regional measurement of three-dimensional wall curvedness and thickness and hence permit accurate estimate of diastolic and systolic wall stress assessment. The entire process taking about 20 min per subject would garner its wider application in clinical practice.

### Wall stress, curvature and curvedness in hypertrophic cardiomyopathy

HCM implies a higher-than-normal myocardial mass, with a high ratio of ventricular wall thickness to radius (*h*/*R*). Based on the different pattern of hypertrophy, systolic and diastolic wall stress were proposed as a stimulus for replication of cardiomyocytes and cardiac remodeling. Indeed, HCM has been reported to correlate with ratio of *h*/*R* or *h*/*R*^3^ or volume/mass from the previous studies (Petersen et al., 2005). This phenomenon allows the preservation of endocardial motion despite reduced shortening of individual fibers such that the EF remains normal (de Simone and Devereux, 2002). On the other hand, progressive LV remodeling in HCM contributes to a change in wall curvature or curvedness (Reant et al., 2015). Our diastolic wall stress, based on ratio of *h*/*R* which consider regional three-dimensional wall curvedness represents an integrated assessment and permit more accurate regional assessment of stress state. These studies provide a rationale supporting wall stress as an ideal index for assessing HCM. Moreover, multivariate stepwise selection analysis identified our wall stress index as the best single predictor of HCM group, and had better sensitivity than LVEF, area strain and wall thickness from both 2D CMR clinical and 3D model measurements.

Our analysis of wall curvedness, stress and function in this study add further insight of subtype of HCM (i.e., sigmoid, reverse and neutral subtypes). The data in present study demonstrated that only reverse curvature HCM subtype presented abnormal wall stress and area strain despite its preserved ejection fraction. This observation is consistent with the finding of Kobayashi et al. in patients with obstructive HCM (Kobayashi et al., 2014). They found that patients with the reverse curvature subtype had less global longitudinal systolic and diastolic strain than patients with sigmoid and concentric hypertrophy despite being younger and less hypertensive. As suggested by Binder (Binder et al., 2006), the reverse curvature morphological subtype may inherently precede and incite the myocyte and fiber disarray and local wall stress perturbations, which are characteristic of HCM.

### Clinical implication

Understanding of LV wall stress may help to solve some clinical questions like the differentiation of adaptive and maladaptive hypertrophy in HCM. At the early stage, both diastolic and systolic wall stresses are maintained “normal” because increased wall thickness is counterbalancing the elevated ventricular pressure. Progressively, wall stress continues to decreases, which causes increase of wall curvature and decrease of the wall radius. These constitute maladaptive hypertrophic developments. We believe our comprehensive suite of quantitative regional curvedness-based wall stress can distinguish early remodeling in HCM and facilitate personalization of monitoring of the natural disease progression or treatment response. For instance, 3D regional parameters derived from our approach may be used to quantify the efficacy and effects (e.g., wall stress) of septal myectomy or ablation therapies in HCM.

### Limitations of study

Limitations of the present study are summarized as follows. First, our approach to 3D LV reconstruction relies on manual delineation of endocardium and epicardium contours obtained from CMR images. This is time consuming and may be replaced by automatic segmentation techniques (Petitjean and Dacher, 2011; Kang et al., 2012; Yang et al., 2016).

Second, curvature-based ventricular wall stress computation depends upon image quality and accuracy of reconstructed surface. Image quality and resolution can be improved by utilizing a 3.0 Tesla scanner rather than a 1.5 Tesla scanner, thereby increasing the quality of the input data for 3D mesh reconstruction and processing. In clinical practice, the spacing between two consecutive CMR short-axis image slices is typically 5–10 mm. Hence, interpolation is used to reconstruct the surface between slices. This process of interpolation may affect the accuracy of the wall stress computation. It should be noted that the intra- and inter- observer variation is small (i.e., both <7%) for wall stress determination, suggesting that our approach is reasonable, and unaffected by variations in the interpolation process.

## Author contributions

Conception or design of the work: XZ, S-KT, YS, MW, SL, J-MZ, and LZ; Acquisition of data for the work: R-ST, and HT; Analysis, interpretation of the work: XZ, R-ST, S-KT, YS, and LZ; Draft the work or revise it critically for important intellectual content: XZ, R-ST, H-CT, S-KT, YS, JA, GK, and LZ; All authors have seen and approved the final version of manuscript.

### Conflict of interest statement

The authors declare that the research was conducted in the absence of any commercial or financial relationships that could be construed as a potential conflict of interest.
